# Epstein Barr Virus Associated B-Cell Lymphomas and Iatrogenic Lymphoproliferative Disorders

**DOI:** 10.3389/fonc.2019.00109

**Published:** 2019-03-07

**Authors:** Jennifer L. Crombie, Ann S. LaCasce

**Affiliations:** Department of Medical Oncology, Dana-Farber Cancer Institute, Boston, MA, United States

**Keywords:** PTLD, post-transplant lymphomatous disorder, ebv, Epstein-Barr virus, lymphoma, DLBCL, Burkitt leukemia/lymphoma (BL)

## Abstract

Epstein-Barr virus (EBV) is a ubiquitous herpesvirus, affecting up to 90% of the population. EBV was first identified as an oncogenic virus in a Burkitt lymphoma cell line, though subsequently has been found to drive a variety of malignancies, including diffuse large B-cell lymphoma (DLBCL) and other lymphoma subtypes. EBV has a tropism for B-lymphocytes and has the unique ability to exist in a latent state, evading the host immune response. In cases of impaired cell mediated immunity, as in patients with advanced age or iatrogenic immune suppression, the virus is able to proliferate in an unregulated fashion, expressing viral antigens that predispose to transformation. EBV-positive DLBCL not otherwise specified, which has been included as a revised provisional entity in the 2016 WHO classification of lymphoid malignancies, is thought to commonly occur in older patients with immunosenescence. Similarly, it is well-established that iatrogenic immune suppression, occurring in both transplant and non-transplant settings, can predispose to EBV-driven lymphoproliferative disorders. EBV-positive lymphoproliferative disorders are heterogeneous, with variable clinical features and prognoses depending on the context in which they arise. While DLBCL is the most common subtype, other histologic variants, including Burkitt lymphoma, NK/T-cell lymphoma, and Hodgkin lymphoma can occur. Research aimed at understanding the underlying biology and disease prevention strategies in EBV-associated lymphoproliferative diseases are ongoing. Additionally, personalized treatment approaches, such as immunotherapy and adoptive T-cell therapies, have yielded encouraging results, though randomized trials are needed to further define optimal management.

## Introduction

EBV is a ubiquitous human herpesvirus, affecting up to 90% of the population depending on the region (1). EBV was the first oncogenic virus identified, initially identified in association with a Burkitt lymphoma (BL) cell line, though subsequently associated with a variety of malignancies, including a variety of lymphoma subtypes (2).

EBV initially infects epithelial cells of the oropharynx, prior to replication and spread to B-lymphocytes. A key feature that provides oncogenic potential is the capacity of the virus to exist in a latent state within B-cells (3, 4). One theory of EBV pathogenesis involves the transit of infected B-cells through the germinal center, where they develop into resting memory B-cells, thus allowing the virus to remain quiescent and occasionally reactivating to infect new B-cells (5). Latent EBV subsequently predisposes to malignant transformation, especially in the setting of impaired cell mediated immunity and chronic antigenic activation, where the virus replicates and expresses viral antigens that promote growth and survival of the cell.

Depending on the viral gene expression pattern, EBV establishes one of three latency patterns.

Latency type I, in which Epstein-Barr nuclear antigen I (EBNA-1) and two small non-coding Epstein-Barr- encoded RNAs (EBERs) are expressed, is generally thought to be associated with BL (3). In latency type II, the virus expresses EBNA-1, EBERs and the latent membrane proteins (LMPs), LMP-1, LMP-2A, and LMP-2B (3). This latency pattern has been associated with Hodgkin lymphoma (HL) and T-cell non-Hodgkin lymphomas. Lastly, latency type III results in expression of the entire EBV repertoire, including EBNAs, EBERs, and LMPs, and is seen in association with post-transplant lymphoproliferative disorders (PTLDs) and other immunocompromised states (3, 6). EBV-positive (EBV+) diffuse large B-cell lymphoma (DLBCL) is associated with both latency type II and III patterns (7). EBV latency proteins play a critical role in disrupting key signaling pathways that promote lymphomagenesis. LMP-1, for example, can behave as an oncogene leading to constitutive signaling, B-cell activation, and upregulation of anti-apoptotic proteins (8). EBNAs can similarly serve as transcription factors, promoting the growth and transformation of B-cells (8).

EBV has been associated with the pathogenesis of a variety of malignancies, most notably B-cell and T-cell lymphomas. In this review, we will discuss the B-cell lymphoproliferative disorders associated with EBV, which often occur in the setting of immune suppression. We will highlight the defining clinical characteristics of these lymphoma subtypes, predisposing and prognostic factors, and general treatment algorithms. Importantly, we will highlight recent advances in treatment approaches, including the application of immune-based and cellular therapies, that have potential to be paradigm shifting in the management of EBV-associated disease independent of histologic subtype.

## Burkitt Lymphoma

EBV was first identified in association with a BL cell line (2). BL is an aggressive B-cell non-Hodgkin lymphoma (NHL), that can be classified into three distinct variants. Endemic BL primarily affects children in equatorial Africa and is nearly universally associated with EBV infection. Alternatively, sporadic BL, which occurs worldwide, is a rarer subtype of NHL that is rarely associated with the virus. The third variant, immunodeficiency-related BL commonly occurs in the context of human immunodeficiency virus (HIV) infection. The role of EBV in this subtype is less clear than in endemic BL, though is seen in up to 40% of cases (9).

Across subtypes, BL is characterized pathologically by monomorphic medium-sized cells with a proliferation index of nearly 100%. There is also a resulting “starry sky” appearance due the presence of numerous tangible body macrophages, phagocytosing abundant apoptotic debris (10). BL typically expresses pan-B-cell antigens, including CD19, CD20, and CD79b and co-expresses CD10 and BCL-6. A defining feature is the presence of translocation between the *c-MYC* oncogene and the immunoglobulin heavy chain (*IgH*) or in rarer cases, the immunoglobulin light chain gene. MYC subsequently serves as a master regulator of oncogenesis, promoting proliferation, apoptosis, differentiation, and metabolism (11).

Endemic BL accounts for approximately half of pediatric malignancies and up to 90% of lymphoma diagnoses in equatorial Africa (9). These lymphomas primarily present as isolated masses of the jaw and occur with a male predominance. Interestingly, endemic BL is restricted to geographic regions in which *Plasmodium falciparum* malaria is holoendemic, suggesting that EBV and malaria infections cooperate in the predisposition of BL. It has been proposed that malaria has immunostimulatory effects on B-cells and results in impairment of T-cell immunity, allowing for selective activation of EBV-infected memory B-cells. One proposed mechanism is the binding of *P. falciparum* to toll-like receptor-9, which can induce enzyme activation-induced cytidine deaminase, thus predisposing to genomic instability (9, 12)

While sporadic BL is highly treatable with intensive chemotherapy, access to high-intensity therapy is often limited for patients with endemic disease (13–23) (Table 1). A variety of chemotherapy regimens, including those which are low and high intensity, are used throughout sub-Saharan Africa. Outcomes for these patients are unfortunately much worse than in resource-rich countries, with overall survival ranging from 51 to 67% (24).

**Table 1:** Treatment options for patients with Burkitt Lymphoma.

**Treatment**	**EFS/PFS/CCR**	**OS**
CODOX-M/IVAC	2-year EFS: 92% (15) 2-year EFS: 65% (16) 2-year PFS: 64% (17)	2-year OS: 73% (16) 2-year OS: 67% (17)
GALGB Regimen	3-year EFS: 52% (cohort 1), 45% (cohort 2) (18) 3-year EFS: 74% (19)	3-year OS: 54% (cohort 1), 50% (cohort 2) (18) 2-year OS: 78% (19)
HyperCVAD +/–R	3-year CCR: 61% (20) 3-year EFS: 80% (21)	3-year OS: 49% (20) 3-year OS: 89% (21)
Dose-adjusted R-EPOCH	EFS: 95% (14)	OS: 100% (14)

*CODOX-M/IVAC, cyclophosphamide, vincristine, doxorubicin, methotrexate, ifosfamide, etoposide, cytarabine*.

*CALGB, Cancer and Leukemia Group B*.

*CVAD, cyclophosphamide, vincristine, doxorubicin, dexamethasone, rituximab*.

*R-EPOCH, rituximab, etoposide, prednisone, vincristine, cyclophosphamide, doxorubicin; EFS/PFS/CCR, event-free survival/progression-free survival/continuous complete response; OS, overall survival*.

## EBV-Positive Diffuse Large B-Cell Lymphoma, NOS

In addition to its association with BL, EBV has been linked to other lymphoma subtypes, including DLBCL. In 2003, EBV+ DLBCL was first described as a distinct entity among elderly patients with *in situ* hybridization demonstrating an association with EBV (25). LMP1 was also detected in all cases described (25). EBV-associated DLBCL of the elderly was subsequently included as a provisional entity in the 2008 World Health Organization (WHO) classification of lymphoid malignancies, defined as a monoclonal large B-cell proliferation occurring in patients over the age of 50 and without known immunodeficiency or history of lymphoma. This disease was thought to occur in setting of immunosenescence, given shared features with post-transplant lymphoproliferative disorder (PTLD) (26). More recently, this entity has been appreciated in younger, immunocompetent hosts, without an association with poor outcome (27, 28). This information has led to the revised entity, EBV+ DLBCL not otherwise specified (NOS) in the 2016 WHO criteria (29).

Pathologically, EBV+ DLBCL generally demonstrates a diffuse and polymorphic proliferation of large lymphoid cells with a varying degree of reactive components such as small lymphocytes, plasma cells, histiocytes, and epithelioid cells (30). Two morphologic variants, monomorphic and polymorphic, have been recognized, though the prognostic significance of these subtypes is not clear. Malignant cells express CD19, CD20, CD22, and CD79. CD30 is expressed in 40% of cases. Most cases are of activated B-cell (ABC) subtype, expressing MUM1/IRF4 and staining negative for CD10 and BCL-6. EBV latent membrane protein 1 (LMP1) is expressed in approximately two-thirds of cases, which is suggestive of EBV latency type II (32) (Table 2). EBV nuclear antigen 2 (EBNA2), which denotes EBV latency type III, typically comprises the remaining one-third (32).

**Table 2:** Epstein-Barr virus latency patterns.

**Latency pattern**	**EBV gene expression pattern**	**EBV-associated lymphoma**
Type I	EBNA-1 EBERs	Burkitt Lymphoma
Type II	EBNA-1 EBER LMPs: LMP-1, LMP-2A, and LMP-2B	Hodgkin lymphoma T-cell NHL EBV+ DLBCL
Type III	Entire EBV repertoire: including EBNAs, EBERs, and LMPs	PTLD EBV+ DLBCL

*EBV, Epstein-Barr Virus; EBNA, Epstein-Barr nuclear antigen; EBER, Epstein-Barr-encoded RNA; LMP, latent membrane proteins*.

As the original name suggests, EBV+ DLBCL is more common in elderly patients, and occurs with a male predominance. It is thought to be associated with poor outcomes as compared to EBV-negative (EBV–) counterparts, though data is conflicting. A Japanese study demonstrated that EBV positivity was associated with an age >60 years, advanced stage, more than one extranodal site of involvement, higher International Prognostic Index (IPI) risk score, presence of B-symptoms, and poorer outcome in response to initial treatment, as compared to EBV– controls (33). This translated to a significantly poorer overall survival of 35.8 months in the EBV+ group vs. an overall survival that was not reached in the EBV– group (33). It should be noted that patients in this study were treated with CHOP (cyclophosphamide, doxorubicin, vincristine, and prednisone) chemotherapy and had an EBV-encoded RNA (EBER) cutoff of 20% (33). Another study demonstrated that age >70 years and the presence of B-symptoms were independent predictors of survival in this disease, thus defining a distinct prognostic model to define three risk groups (30). In Western countries, in which rituximab was added to a CHOP chemotherapy backbone (R-CHOP) and EBV positivity was defined by EBER >10% of cellular staining, there were no appreciable differences between the clinical characteristics of EBV+ and EBV– disease, however, CD30 positivity in conjunction with EBV positivity, was found to confer an inferior prognosis (32). As a cutoff for EBV positivity has not been defined and treatment has not been uniform, the impact of EBV positivity on outcome remains to be determined. Differences may also be attributable to variation in EBV stains or host factors across geographic regions.

Despite conflicting reports on clinical and prognostic features, EBV+ DLBCL does have a unique molecular phenotype as compared to EBV-negative disease. Specifically, gene expression profiling demonstrates that EBV+ DLBCL has a molecularly distinct profile (7). Specifically, gene expression profiling of EBV+ DLBCL demonstrated upregulation of genes involved in NF-κB activity, cell proliferation, cell-cycle progression, and cell metabolism. Immunohistochemistry has also identified increased expression of p50 and pSTAT3, components of NF-κB signaling, in EBV+ DLBCL (32). While these differences have been appreciated, the prognostic and therapeutic implications of these distinct molecular features remain unclear.

The standard of care for frontline therapy remains treatment with chemoimmunotherapy, traditionally R-CHOP, with variable responses reported worldwide. Emerging treatment strategies, including viral directed approaches and use of immune-based therapies are an active area of investigation and are discussed in further detail below. While these therapies have primarily been studied in the context of post-transplant lymphoproliferative disorder (PTLD), they have broad implications across EBV-driven lymphomas such as EBV+ DLBCL NOS.

## Iatrogenic Lymphoproliferative Disorders Associated With EBV: Transplant and Non-Transplant Settings

### Post-transplant Lymphoproliferative Disorder

PTLD is a serious complication of solid organ and allogenic stem cell transplantation. With the exception of skin cancer and *in situ* cervical cancer, PTLD is the most common malignancy seen after solid organ transplantation (34). The risk of PLTD is dependent on a variety of factors, including the type transplant, the immunosuppression used, and EBV seronegativity of the recipient prior to transplant. Following solid organ transplant, PTLD is thought to be derived from recipient lymphoid cells, while PTLD following hematopoietic stem cell transplantation are almost exclusively of donor origin (35, 36). Over the past 10–15 years, there has been an increase in the incidence of PTLD, presumably due to an increased number of organ transplants performed, use of novel immunosuppressive therapies, and an increased awareness of the disease (37, 38).

The WHO classifies PTLD into six categories, with three types of non-destructive PTLDs, including plasmacytic hyperplasia, infectious mononucleosis-like PTLD, and florid hyperplasia, as well as polymorphic lesions, monomorphic lesions, and classical HL (29, 38, 39) (Table 3). The majority of PTLDs are of B-cell origin and driven by EBV, though EBV– cases have been reported in 10–48% of cases (40). As T-cell responses are impaired in the post-transplant setting, EBV is able to promote B-cell proliferation and potentially transformation in an unregulated fashion. These lymphomas, like other lymphomas arising in the setting of immunosuppression, typically express a latency type III program. The pathogenesis of EBV– PTLD is less clearly understood. PTLD is most commonly seen within the 1st year following transplantation, though a subset of PTLD, especially those that are EBV– can occur late. As gene expression profiling reveals biologically distinct categorization among EBV+ and EBV– disease, it has been raised whether EBV– PTLD represents lymphoma that coincidentally occurs in the setting of transplantation, though additional analyses dissecting biologic and clinical differences between these entities are required (40, 41). The most common subtype of monomorphic PTLD is DLBCL, comprising ~60% of cases in one study, though other histologies including BL, plasma cell neoplasms and T-cell lymphomas have been seen (34, 42). Classical HL-type PTLD is very rare, though can occur, especially late after transplant (43).

**Table 3:** WHO classification of post-transplant lymphoproliferative disorders.

**PTLD category**	**Type**	**EBV status**
Early lesions (Non-destructive)	Plasmacytic hyperplasia Infectious mononucleosis-like PTLD Florid hyperplasia	Almost 100%
Polymorphic (Destructive)	Polyclonal and Monoclonal proliferations	>90%
Monomorphic (Destructive)	Monoclonal Non-Hodgkin Lymphomas, including: - Diffuse large B-Cell lymphoma (~60%) - Burkitt lymphoma - Plasma cell myeloma - T-cell lymphoma	Both EBV+ and EBV–(EBV– in 10–48% of cases)
Hodgkin lymphoma (Destructive)	Monoclonal	>90%

### Risk Factors

With both solid organ and hematologic stem cell transplants, the risk of PTLD is dependent on the type of transplant performed. In adults, the risk is highest following multi-organ and intestinal transplants (>20%), followed by lung transplants (3–10%), heart transplants (2–8%), liver transplants (1–5.5%), pancreatic transplants (0.5–5%), and kidney transplants (0.8–2.5%) (34, 38, 42, 44) (Table 4). In addition to variation in histocompatibility among organ types and requirements for immunosuppression, transplanted organs can have varying degrees of EBV+ lymphoid tissue within the graft, which contribute to the variation in risk (45). In the case of allogeneic stem cell transplant, the risk of PTLD risks appears to be associated with the degree of HLA mismatch, with highest rates of PTLD occurring after unrelated or HLA-mismatched grafts (46, 47). With the advent of haploidentical transplantation, the incidence of PTLD has further increased, though this risk may be abrogated with the use of post-transplant cyclophosphamide for prophylaxis against graft vs. host disease (GVHD) (48).

**Table 4:** Risk factors for PTLD (38).

**Solid organ transplant**	**Allogeneic stem cell transplantation**
**Type of transplant**	**Degree of HLA mismatch**
- Multi-organ and intestinal transplants: >20% - Lung transplants: 3–10% - Heart transplants: 2–8% - Liver transplants: 1–5.5% - Pancreatic transplants: 0.5–5% – Kidney transplants: 0.8–2.5%	- Haploidentical transplants: ≤20% - Unrelated donor: 4–10% - Umbilical cord transplant: 4–5% - HLA-identical related: 1–3%
**Immunosuppressive therapy**	**Type of GVHD prophylaxis**
- Higher intensity and prolonged duration of therapy associated with higher risk - Use of Anti-thymocyte globulin (ATG), Calcineurin inhibitors, Anti-CD3 (OKT3), Tacrolimus, Cyclosporine	- T-cell depletion associated with highest risk
**EBV mismatch between recipient and donor**	**EBV mismatch between recipient and donor**
- Relative risk between 10 and 75	

The degree, duration and type of immunosuppression are also major factors that impact the risk of PTLD. Several studies have demonstrated rising rates of PTLD in the setting of more potent immunosuppression (42, 44, 49). Although it can difficult to identify the specific contribution of one drug within a combination immunosuppressive regimen, anti-thymocyte globulin (ATG), calcineurin inhibitors, anti-CD3 (OKT3), tacrolimus, and cyclosporine have all been specifically implicated in PTLD predisposition, the risks can vary across studies (49). In one study of 200,000 kidney recipients, immunosuppression with cyclosporine did not confer added risk compared with azathioprine/steroid treatment, whereas treatment with tacrolimus increased the risk ~2-fold (44). In this study, OKT3 or ATG were also been found to significantly increase lymphoma rates. Patients treated with tacrolimus also been found to have an increased risk of PTLD as compared to those treated with cyclosporine and antimetabolites, mycophenolate and azathioprine, in other studies (50). Following allogeneic transplant, the type of GVHD prophylaxis also appears to contribute to PTLD risk, with T-cell depletion being associated with highest incidence of lymphoma (46).

EBV mismatch between recipient and donor is the strongest prognostic factor for the development of PTLD. In one study, EBV mismatch among renal transplant donor and recipients (donor positive/recipient negative) was associated with a 35–42% increase in the incidence of PTLD (51). A similar study among solid organ transplant recipients demonstrated a 24-fold increased risk of PTLD among EBV seropositive patients (52). Interestingly, CMV seronegativity further amplifies risk, suggesting that CMV viremia may further predispose to EBV viremia. Increased EBV naivety in children as compared to adults is also thought to contribute to the increased risk of PTLD in the pediatric population, where primary EBV infections have the potential to drive lymphomagenesis (38).

### Prognosis

PTLD has traditionally been associated with poor outcomes, though survival rates have improved with the incorporation of early rituximab-based therapy (53). While disease presentations can be heterogeneous, patients are frequently diagnosed with advanced stage disease with extranodal involvement, including high rates of graft and gastrointestinal disease. In one study, 72% of patients were diagnosed with Ann Arbor stage III/IV disease and 78% of patients displayed one or more extranodal sites of disease (42). In addition to the IPI, the presence of CNS and bone marrow involvement, as well as hypoalbuminemia, have been shown to have prognostic value (53). A multivariable model for survival constructed using three factors: poor performance status, monomorphic disease, and graft organ involvement, has also had superior performance to the IPI in separating survival outcomes of patients, though additional prospective studies are required to validate the use of this tool (54). A French study, which included 500 renal transplant recipients with PTLD, similarly demonstrated that advanced age, elevated creatinine, elevated lactate dehydrogenase, disseminated lymphoma, brain localization, invasion of serous membranes, monomorphic PTLD, and T-cell PTLD were independent prognostic indicators of poor response (55). While the resulting prognostic score effectively stratifies risk, it remains unclear whether it provides additional prognostication outside of traditional models such as the IPI (56). More recently, risk adaptive therapy has been utilized for the management of PTLD, with response to single agent rituximab induction serving as a prognostic factor for overall survival (31).

### Treatment

Reduction in immunosuppression is the mainstay of initial therapy for patients with PTLD. While this treatment approach must always be weighed with the risk of graft rejection, studies have demonstrated that reduction of immune suppression alone can result in partial PTLD regression in a large subset of patients with both polymorphic and monomorphic disease (57). This typically involves reduction in calcineurin inhibitors by at least 50% and discontinuation of azathioprine or mycophenolate mofetil (58). Following allogenic transplant, reduction in immunosuppression is defined as a sustained decrease of at least 20% of the daily dose of immunosuppressive drugs with the exception of low-dose corticosteroid therapy (59). Early disease stage predicts favorable responses to reduction in immunosuppression, while older age, bulky disease and advanced stage are associated with poor responses with this strategy (57). While responses have been reported in monomorphic disease, patients invariably require addition therapy for adequate disease control.

**Figure 1:**
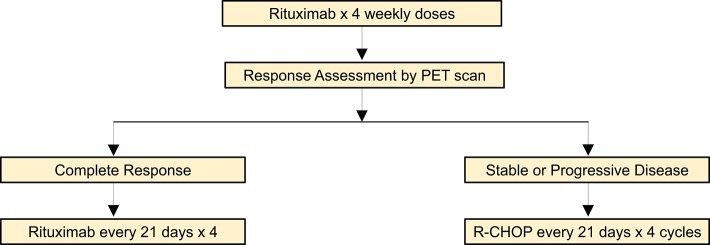
Risk-adaptive treatment approach for DLBCL PTLD (31).

In addition to tapering of immune suppression, rituximab with or without chemotherapy is an effective therapeutic option in PTLD. The phase II PTLD-1 trial initially investigated the efficacy of 4 weekly doses of rituximab followed by four cycles of CHOP chemotherapy in adult solid organ transplant recipients with CD20+ PTLD (60). Interestingly, this study demonstrated that 60% of patients had a complete or partial response to rituximab monotherapy prior to the initiation of chemotherapy. The high responses to single agent rituximab prompted a follow up phase II study to assess whether rituximab consolidation was an adequate treatment approach for patients with PTLD who achieved a complete response to rituximab induction (31). In patients who did not achieve a complete response, treatment proceeded with four additional cycles of rituximab plus CHOP, as in the PTLD-1 trial. This treatment approach resulted in an overall response rate of 88% and median overall survival of 6.6 years, which were similar to outcomes of patients receiving chemotherapy (Figure 1). While risk stratified sequential therapy is an appropriate strategy for DLBCL PTLD, disease specific treatment approaches are utilized for other histologic subtypes.

The expression of viral antigens makes EBV+ PTLD an attractive candidate for immune based therapies. EBV-specific cytotoxic lymphocytes have been successfully utilized following allogeneic stem cell transplant, where EBV+ PTLD is known to originate from donor lymphocytes, thus reconstituting T-cell immunity against the virus. One study demonstrated that infusion of EBV-specific cytotoxic T-lymphocytes (CTLs) generated from the transplant donor both prevented and treated PTLD in the post allogeneic stem cell setting (61). In this study none of 110 patients who used CTLs for prophylaxis developed PTLD and 11 of 13 patients with PTLD achieved a complete remission (61). Similarly, autologous CTLs have been successfully utilized for prevention of PTLD in solid organ transplant recipients, in which PTLD is primarily thought to be derived from recipient lymphocytes, though the length of persistence and antiviral activity of the CTLs does not appear as robust as in allogenic stem cell transplant recipients (62). Partially HLA-matched CTLs have subsequently been identified as an option for solid organ transplant recipients, resulting in an overall response rate of 64% at 5 weeks, though are associated with prolonged production times (63). Autologous T cells directed to the LMP2 or LMP1 and LMP2 antigens have further demonstrated promising results in patients with a range of EBV+ lymphomas (64). Additional prospective studies will be required to establish the feasibility and efficacy of these approaches in a larger patient cohorts.

Data has suggested that EBV-associated malignancies, such as PTLD and classical HL, share certain mechanisms of immune evasion (65, 66). Therefore, there has been interest in utilizing immune checkpoint inhibitors in PTLD, given known efficacy in cHL (67). Programmed cell death ligand 1 (PD-L1) is expressed on antigen-presenting cells that bind the PD-1 receptor on T cells, thus inhibiting T-cell receptor signaling. Recent data suggests that EBV plays a role in increasing PD-L1 in PTLD, further supporting the role of checkpoint inhibition in this disease (66). PDL-1, PDL-2, and PD-1 were also found to be positive in 67% of PTLD cases following solid organ transplantation in one study (68). While checkpoint inhibitors have been utilized in the setting of both solid organ and allogeneic stem cell transplant, additional studies will be required to determine the safety, efficacy and risk of graft rejection or graft vs. host disease with this approach.

### Prevention

The role of antiviral medications for the prevention of PTLD has remained controversial. A recent meta-analysis found no significant difference in the rate of EBV+ PTLD in solid organ transplant recipients among those who received prophylaxis, including acyclovir, valacyclovir, ganciclovir, valganciclovir, compared with those who did not receive prophylaxis (69). While these therapies are moderately effective in suppressing viral replication and shedding during acute or lytic replication, they are not effective against EBV given the latent state of replication (70). Vaccines against EBV, specifically those targeting pg350, have thus far been unsuccessful, though the identification of alternative epitopes for vaccine generation are underway (71). Pre-emptive therapy with rituximab in patients with positive EBV DNA following allogenic stem cell transplantation has also been associated with reduction in PTLD incidence and abrogation of PTLD-related mortality (72, 73). Pre-emptive strategies including tapering of immune suppression and rituximab therapy are also used following solid organ transplant, though practices appear less uniform (74).

### Iatrogenic Immunodeficiency-Associated Lymphoproliferative Disorder-Non-transplant Related

Lymphoproliferative disorders are known to occur frequently in the context of impaired immunity. As such, patients outside of the transplant setting, including those using immunomodulatory agents, are at increased risk for lymphoproliferative disorders, especially EBV-driven disease. The WHO defines a subset of “other iatrogenic immunodeficiency associated lymphoproliferative disorders,” composed primarily of lymphomas occurring in patients receiving immunosuppressive therapy for autoimmune disease. In particular, EBV+ lymphoproliferative disorders have been seen in patients on methotrexate, tumor necrosis factor (TNF)α inhibitors, fludarabine and mycofenolate mofetil (75–78). The presence of an underlying autoimmune disease may also play a role in disease pathogenesis, though the association remains less clear. One study of rheumatoid arthritis associated lymphoproliferative disorders, demonstrated a range of lymphoid histologies, including DLBCL, classical HL, polymorphic B-cell lymphoproliferative disorder, follicular lymphoma, composite lymphoma, reactive lymphadenitis, and peripheral T-cell lymphoma (75). Of note, EBV was positive in 60% of patients and withdrawal of methotrexate resulted in disease regression in 59% of cases, suggesting similar characteristics to PTLD (75). While treatment algorithms are less clearly defined in these lymphoproliferative disorders as compared to PTLD, treatment typically consists of standard of care therapies for the specific histology seen.

## Conclusions

EBV is a common herpesvirus that has a unique ability to evade the host immune response and exist in a latent form within B-lymphocytes. Impairments in cellular immunity, occurring in the setting of concurrent infections, older age, solid organ, and hematopoietic stem cell transplantation, and immunosuppressive therapies, results in a particularly high risk of EBV reactivation and B-cell transformation. EBV-associated lymphoproliferative disorders have heterogeneous presentations with variable responses to therapy. However, given a shared underlying biology and overlapping clinical features, adaptations to the current classification structure of immunodeficiency-associated lymphoproliferative disorders have been proposed (79). Ongoing research aimed at novel treatment approaches, especially those utilizing immune-based therapies have demonstrated promising results in PTLD, and have implications across EBV-driven histologies. Ongoing research aimed at understanding the biology of EBV as well as disease prevention and treatment strategies will be fundamental in improving outcomes for patients with EBV-associated lymphoproliferative diseases.

## Data Availability

All datasets generated for this study are included in the manuscript and/or the supplementary files.

## Author Contributions

All authors listed have made a substantial, direct and intellectual contribution to the work, and approved it for publication.

### Conflict of Interest Statement

The authors declare that the research was conducted in the absence of any commercial or financial relationships that could be construed as a potential conflict of interest.
